# Elucidating Direct Photolysis Mechanisms of Different Dissociation Species of Norfloxacin in Water and Mg^2+^ Effects by Quantum Chemical Calculations

**DOI:** 10.3390/molecules22111949

**Published:** 2017-11-11

**Authors:** Se Wang, Zhuang Wang

**Affiliations:** Collaborative Innovation Center of Atmospheric Environment and Equipment Technology, Jiangsu Key Laboratory of Atmospheric Environment Monitoring and Pollution Control, School of Environmental Science and Engineering, Nanjing University of Information Science and Technology, Nanjing 210044, China

**Keywords:** norfloxacin, direct photolysis pathways, DFT, ionic forms, Mg^2+^

## Abstract

The study of pollution due to combined antibiotics and metals is urgently needed. Photochemical processes are an important transformation pathway for antibiotics in the environment. The mechanisms underlying the effects of metal-ion complexation on the aquatic photochemical transformation of antibiotics in different dissociation forms are crucial problems in science, and beg solutions. Herein, we investigated the mechanisms of direct photolysis of norfloxacin (NOR) in different dissociation forms in water and metal ion Mg^2+^ effects using quantum chemical calculations. Results show that different dissociation forms of NOR had different maximum electronic absorbance wavelengths (NOR^2+^ < NOR^0^ < NOR^+^) and showed different photolysis reactivity. Analysis of transition states (TS) and reaction activation energies (*E*_a_) indicated NOR^+^ generally underwent loss of the piperazine ring (C10–N13 bond cleavage) and damage to piperazine ring (N13–C14 bond cleavage). For NOR^2+^, the main direct photolysis pathways were de-ethylation (N7–C8 bond cleavage) and decarboxylation (C2–C5 bond cleavage). Furthermore, the presence of Mg^2+^ changed the order of the wavelength at maximum electronic absorbance (NOR^+^-Mg^2+^ < NOR^0^-Mg^2+^ < NOR^2+^-Mg^2+^) and increased the intensities of absorbance peaks of all three dissociation species of NOR, implying that Mg^2+^ played an important role in the direct photolysis of NOR^0^, NOR^+^, and NOR^2+^. The calculated TS results indicated that the presence of Mg^2+^ increased *E*_a_ for most direct photolysis pathways of NOR, while it decreased *E*_a_ for some direct photolysis pathways such as the loss of the piperazine ring and the damage of the piperazine ring of NOR^0^ and the defluorination of NOR^+^.

## 1. Introduction

Antibiotics are widely used in aquaculture, animal husbandry, and medical treatment, due to the fact that they can either kill or inhibit the growth of bacteria [1,2]. Antibiotics are frequently released into the environment due to negligence, and they are frequently detected in the aquatic environment [1,3]. Norfloxacin (NOR) was the first fluoroquinolone antibiotic to be used clinically [4], and has been found to be one of the most frequently detected fluoroquinolone antibiotics in surface waters [5,6,7,8] and municipal wastewater [9]. Recently, a lot of attention has also been devoted to studies on the behavior, fate, and degradation methods of NOR in the aquatic environment [10,11,12].

Photolysis is expected to play an important role in determining the fate and behavior of fluoroquinolone antibiotics including NOR in some sunlit surface waters [13,14,15,16,17,18]. Photolysis includes direct and indirect photolysis. For the direct photolysis of organic pollutants, a molecule itself absorbs photons and is degraded [19]. Indirect photolysis may include reaction with transient excited species such as singlet oxygen, hydroxyl radical, triplet excited state dissolved organic matter, or other reactive species [20]. The UV-Vis absorption spectrum of NOR consists of one major peak at 276 nm and a broad band at approximately 300 to 350 nm [21], which indicates that NOR can undergo direct photolysis in the aquatic environment. The molecular structure of NOR contains ionizable groups (–COOH, –NH_n_), resulting in the aqueous presence of NOR in various dissociation states. Previous studies have indicated that the photolysis rate of NOR is greatly influenced by the pH of the solution [10,11]. However, the mechanisms underlying the direct photolysis of NOR in different dissociation forms have not yet been fully understood.

Recently, issues related to the study of pollution resulting from the combination of antibiotics and metals have been drawing a lot of attention [20,22,23]. Previous studies have suggested that metal ions such as Mg^2+^ can have an effect on the photochemical behavior of organic pollutants [20,24]. For example, Werner et al. [22] found that the direct photolysis rate constant of tetracycline was greatly influenced by the presence of Ca^2+^ or Mg^2+^. Martínez et al. [23] found that metal cations may affect the photochemical properties of NOR. Metal ion Mg^2+^ occurs at a high concentration in natural water environment [25]. Following the environmental release of NOR, there may be interactions between NOR and Mg^2+^. Thus, the mechanisms of Mg^2+^’s effect on the photolysis of different dissociation species of NOR urgently need to be investigated.

Obtaining experimental data can be laborious, costly, and time-consuming. Quantum chemistry calculations have been found to be efficient alternatives for predicting the environmental behavior and fate of organic pollutants, and for providing an important information on reaction intermediates or reactive species involved in chemical reactions that are difficult to be detected experimentally [26,27,28,29,30,31]. It was the purpose of this study to investigate the direct photolysis mechanisms of different dissociation species of NOR in water and Mg^2+^ effects based on density functional theory (DFT).

## 2. Computational Methods

**Figure 1:**
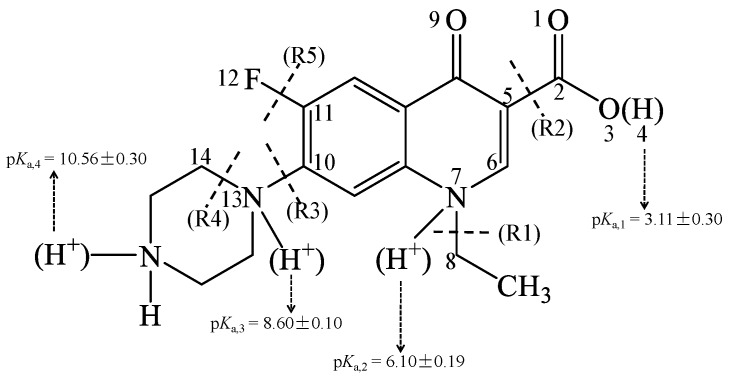
Structural formulae of norfloxacin (NOR) and numbering scheme for atomic positions. The p*K*_a_ values are taken from ref. [41].

NOR was selected as a model compound (Figure 1). The geometry optimization of all structures in solvent water was carried out using DFT [32] and Becke’s three-parameter hybrid exchange function with Lee-Yang-Parr gradient-corrected correlation functional (B3LYP) [33] with 6-311+G (d,p) basis set. The integral equation formalism of polarized continuum model (IEFPCM) was employed to consider the solvent effects in water [34]. The UV absorbance spectra of different ionic forms of NOR and complexes with Mg^2+^ in water were calculated using time-dependent density functional theory (TDDFT) at B3LYP/6-311++G (d,p) level [35,36,37].

The possible direct photolysis reaction pathways were calculated at the lowest excited triplet states, as the lowest excited triplet states have been found to be long-lived photochemical reaction precursors for many compounds [38]. The direct photolysis reaction pathways of different ionic forms of NOR and complexes with Mg^2+^ in solvent water were calculated employing the DFT method at the B3LYP/6-311+G (d,p) level of theory. The geometries at the lowest triplet states were calculated with a spin multiplicity of 3. Frequency calculations were performed at the same level to confirm all the stationary points. Transition states (TS) were characterized with one imaginary vibrational frequency. Intrinsic reaction coordinate (IRC) calculations were performed to confirm that TS do connect with the corresponding reactants and products [39]. Zero-point energy correction was considered for the estimated reaction activation energy. All calculations were carried out using the Gaussian 09 software package (Rev. B. 01; Gaussian Inc.: Wallingford, CT, USA) [40].

## 3. Results and Discussion

### 3.1. Geometries of Three Dissociation Species (*NOR^0^, NOR^+^,* and *NOR^2+^*) in Water

**Figure 2:**
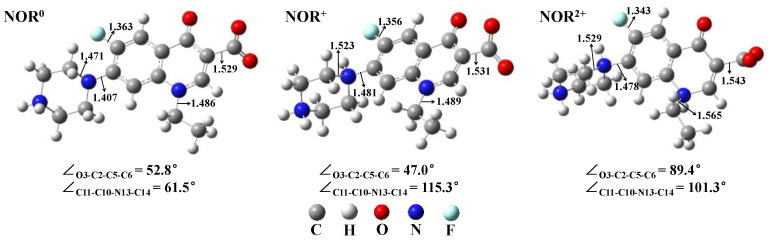
Optimized geometries of three dissociation species of NOR, along with selected bond lengths (Å) and dihedral angles (°).

**Figure 3:**
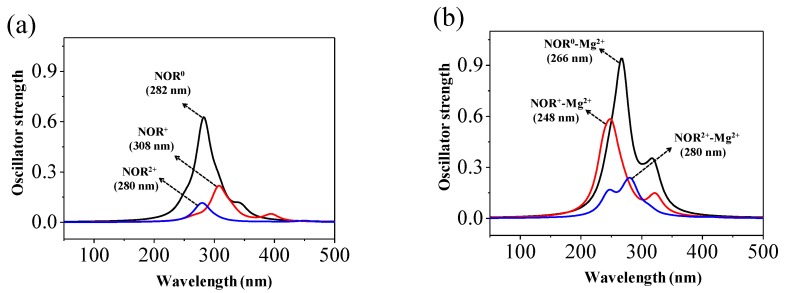
Calculated electronic absorption spectra of (**a**) NOR and (**b**) complexes NOR-Mg^2+^.

NOR may exhibit five dissociation species with p*K*_a,1_ (3.11 ± 0.30), p*K*_a,2_ (6.10 ± 0.19), p*K*_a,3_ (8.60 ± 0.10), and p*K*_a,4_ (10.56 ± 0.30) in water [41], of which three dissociation species NOR^0^, NOR^+^, NOR^2+^ are dominant in natural water environments, and were thus investigated in the present study (Figure 1). The optimized geometries of the three environmentally relevant dissociation species NOR^0^, NOR^+^, and NOR^2+^ are presented in Figure 2. There are differences among the geometries of the three dissociation species, to some extent. For instance, the bond length of N7–C8 of NOR^2+^ (1.565 Å) is longer than that of NOR^0^ (1.486 Å)/NOR^+^ (1.489 Å). The bond length of C10–N13 of NOR^+^ (1.481 Å)/NOR^2+^ (1.478 Å) is longer than that of NOR^0^ (1.407 Å). The bond length of N13–C14 of NOR^+^ (1.523 Å)/NOR^2+^ (1.529 Å) is longer than that of NOR^0^ (1.471 Å). The dihedral angle O3–C2–C5–C6 of NOR^+^ (47.0°) is similar to that of NOR^0^ (52.8°). However, the dihedral angle O3–C2–C5–C6 of NOR^2+^ is 89.4°, indicating that the plane of O3–C2 bond is nearly orthogonal to the plane of C5–C6 bond. Additionally, the computed electronic absorption spectra of the three dissociation forms of NOR in water are shown in Figure 3a. The order of the computed wavelength at maximum electronic absorbance of the three dissociation species in water is NOR^2+^ (280 nm) < NOR^0^ (282 nm) < NOR^+^ (308 nm) at TDDFT/B3LYP/6-311++G (d,p) level. The corresponding experimental data is around 275 nm in water (pH = 7.0) [21] and 274 nm in water (pH = 7.4) [23].

### 3.2. Direct Photolysis Pathways of Three Dissociation Species (*NOR^0^, NOR^+^,* and *NOR^2+^*) in Water

**Figure 4:**
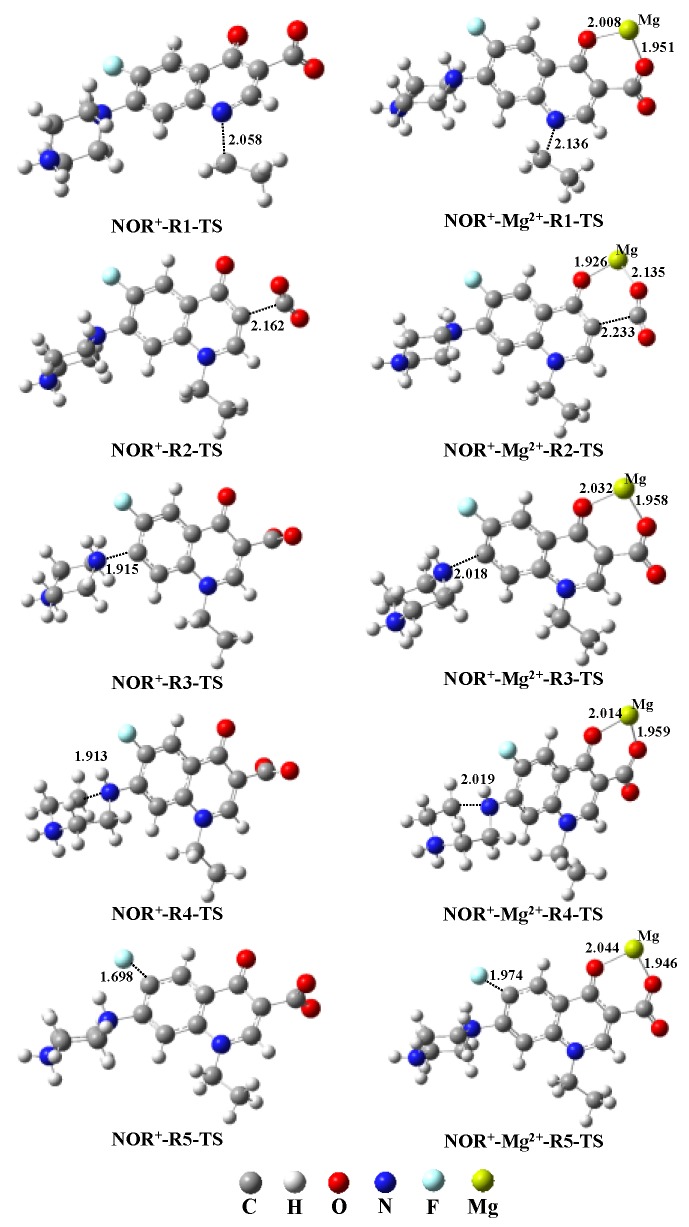
The transition state geometries of direct photolysis pathways R1, R2, R3, R4, and R5 of NOR^+^ and NOR^+^-Mg^2+^.

As depicted in Figure 1, five possible reaction pathways were considered in the calculation of direct photolysis of NOR at the excited triplet states, including de-ethylation (N7–C8 bond cleavage/R1), decarboxylation (C2–C5 bond cleavage/R2), loss of piperazine ring (C10–N13 bond cleavage/R3), damage of piperazine ring (N13–C14 bond cleavage/R4), and defluorination (C11–F12 bond cleavage/R5). The computed reaction activation energies (*E*_a_, kcal·mol^−1^) for the five photolysis reaction pathways in different dissociation forms are listed in Table 1. The optimized geometries of reaction TS are shown in Figure 4 and Figures S1 and S2.

As can be seen from Table 1, the *E*_a_ values of pathway R3 (9.5 kcal·mol^−1^) and R4 (7.7 kcal·mol^−1^) for NOR^+^ are obviously lower than those of other pathways. Thus, pathways R3 and R4 are easier to carry out than other pathways, and are the main pathways for the direct photolysis of NOR^+^. Additionally, the *E*_a_ value of pathway R1 (25.4 kcal·mol^−1^) for NOR^+^ is the highest of all the five photolysis pathways, indicating that the cleavage of the N7–C8 bond (de-ethylation) is the hardest to overcome among the five pathways.

For dissociation form NOR^2+^, the *E*_a_ values of pathway R1 (5.4 kcal·mol^−1^) and R2 (8.9 kcal·mol^−1^) are remarkably lower than those of other pathways (Table 1), revealing that de-ethylation and decarboxylation are the main pathways for the direct photolysis of NOR^2+^. In addition, the *E*_a_ value of pathway R5 is too high (42.5 kcal·mol^−1^) (Table 1), indicating that NOR^2+^ is difficult to defluorinate via the cleavage of the C11–F12 bond. For neutral dissociation form NOR^0^, the *E*_a_ value of pathway R1 (21.0 kcal·mol^−1^) is the lowest, while the *E*_a_ value of pathway R3 (31.2 kcal·mol^−1^) is the highest among all the pathways. Thus, NOR^0^ is difficult to take off the piperazine ring via cleavage of C10–N13 bond. Taken together, NOR^2+^ is the easiest to de-ethylate via the cleavage of the N7–C8 bond among the three dissociation species, and are easier to undergo the cleavage of the C2–C5 bond (decarboxylation) than the other two dissociation species. In addition, NOR^+^ is the easiest with which to carry out the cleavage of the C10–N13/N13–C14 bond among the three dissociation species. Thus, the photochemical reactivities of the different dissociation species of NOR are quite different from each other.

### 3.3. Complex Geometries of Three Dissociation Species (*NOR^0^, NOR^+^,* and *NOR^2+^*) with Metal Ion *Mg^2+^* in Water

**Figure 5:**
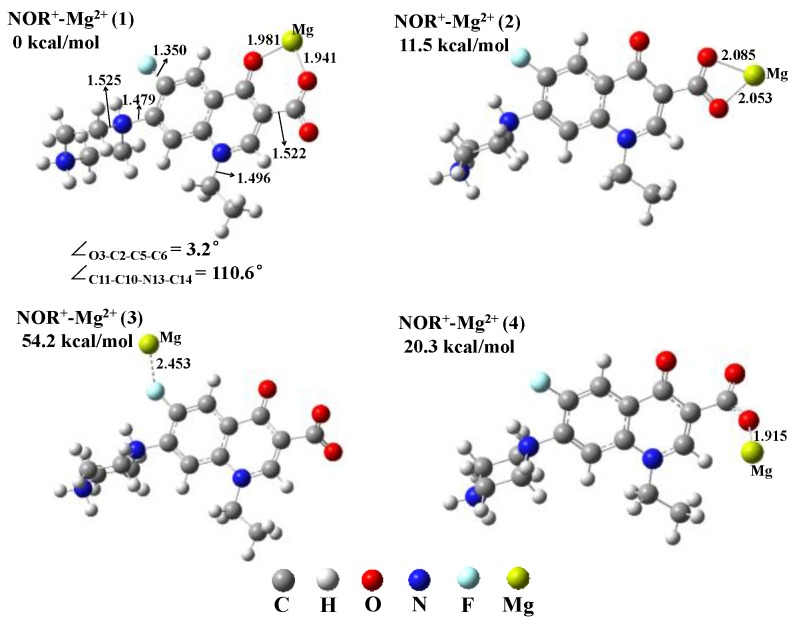
**Four** optimized geometries (1), (2), (3), (4) of complex NOR^+^-Mg^2+^ along with selected bond lengths (Å) and dihedral angles (°). The energies of geometries are relative to that of the most stable geometry NOR^+^-Mg^2+^ (1).

Optimized geometries of the complex NOR-Mg^2+^ are shown in Figure 5 and Figures S3 and S4. Computed results indicate that complex NOR^+^-Mg^2+^ has four possible geometries. NOR^+^-Mg^2+^ (1) is the most stable geometry with the lowest single point energy among the four geometries, and is the main geometry discussed in this research (Figure 5). The results also indicate that NOR^0^-Mg^2+^ (1) and NOR^2+^-Mg^2+^ (1) are the most stable geometries (Figures S3 and S4). All the following calculations are based on the structure NOR^0^-Mg^2+^ (1), NOR^+^-Mg^2+^ (1), and NOR^2+^-Mg^2+^ (1).

The dihedral angle O3–C2–C5–C6 of the monomer NOR^+^ and NOR^0^ is 47.0° and 52.8° (Figure 2), while for complexes with Mg^2+^ the dihedral angle O3–C2–C5–C6 is obviously reduced to 3.2° and 0.0° due to the formation of O1–Mg bond and O9–Mg bond (Figure 5 and Figure S3). In complexes NOR^+^-Mg^2+^ and NOR^0^-Mg^2+^ the O3–C2 bond and C5–C6 bond is almost in a plane. In addition, with the formation of O1–Mg bond and O9–Mg bond in complex NOR^2+^-Mg^2+^, the dihedral angle O3–C2–C5–C6 is also dramatically reduced to 11.9° from 89.4° in monomer (Figure 2 and Figure S4). The plane of the O3–C2 bond is nearly orthogonal to the plane of the C5–C6 bond in monomer NOR^2+^, while the plane of the O3–C2 bond tends to be parallel to the plane of the C5–C6 bond in complex NOR^2+^-Mg^2+^. Additionally, the bond lengths of complexes are also different from those of monomers. For instance, the bond length of N13–C14/N7–C8 in complex NOR^+^-Mg^2+^ is longer than that in monomer NOR^+^, while the bond length of C11–F12/C10–N13/C2–C5 in complex NOR^+^-Mg^2+^ is shorter than that in monomer NOR^+^ (Figure 2 and Figure 5).

The UV absorbance spectra of complexes with Mg^2+^ in water were calculated using TDDFT at B3LYP/6-311++G (d,p) level (Figure 3b). The calculation results show that a blue shift occurs at the maximum electronic absorbance peaks of NOR^0^-Mg^2+^ and NOR^+^-Mg^2+^, compared with that of corresponding monomer. Additionally, Mg^2+^ changes the order of maximum electronic absorbance wavelength of the three dissociation species. The order of maximum electronic absorbance wavelengths of complexes is: NOR^+^-Mg^2+^ (248 nm) < NOR^0^-Mg^2+^ (266 nm) < NOR^2+^-Mg^2+^ (280 nm). Additionally, with the presence of Mg^2+^, there is an increase of 49.9%, 167.3%, and 108.0% in the intensities of absorption peaks for the three dissociation species NOR^0^, NOR^+^, and NOR^2+^, respectively. The experimental results of Martínez et al. [23] also showed that the intensity of maximum absorption peak (274 nm) of NOR at pH = 7.4 is increased by around 42.6% when Mg^2+^ is present. Thus, the metal ion Mg^2+^ has an impact on both the structures and UV absorbance spectra of different dissociation species of NOR, which may also have an impact on direct photolysis of different dissociation species of NOR.

### 3.4. Effects of *Mg^2+^* on Direct Photolysis of Three Dissociation Species (*NOR^0^, NOR^+^,* and *NOR^2+^*) in Water

Five possible reaction pathways were considered in the calculation of direct photolysis of complexes NOR-Mg^2+^ at the excited triplet states, including de-ethylation (N7–C8 bond cleavage/R1), decarboxylation (C2–C5 bond cleavage/R2), loss of piperazine ring (C10–N13 bond cleavage/R3), damage of piperazine ring (N13–C14 bond cleavage/R4), and defluorination (C11–F12 bond cleavage/R5). The computed activation energies (*E*_a_, kcal·mol^−1^) for the five photolysis reaction pathways in different dissociation forms are listed in Table 1. The optimized geometries of reaction TS are shown in Figure 4 and Figures S1 and S2.

For the complex NOR^+^-Mg^2+^, the computed *E*_a_ values of four pathways (R1, R2, R3, and R4) are all higher than those of monomer NOR^+^, indicating that Mg^2+^ hinders the cleavage of the N7–C8 bond, C2–C5 bond, C10–N13 bond, and N13–C14 bond (Table 1). However, Mg^2+^ promotes defluorination via the cleavage of the C11–F12 bond due to the lower *E*_a_ value of reaction R5 compared with that of the monomer. Additionally, Mg^2+^ also changes the main photolysis pathway of NOR^+^. For monomer NOR^+^, the damage of piperazine ring via the cleavage of N13–C14 bond is the easiest to occur due to the lowest *E*_a_ value among all the five pathways. However, for complex NOR^+^-Mg^2+^, the *E*_a_ value of R5 (defluorination) is the lowest among all the five pathways.

For the complex NOR^2+^-Mg^2+^, Mg^2+^ has little impact on the *E*_a_ value of pathway R1, while Mg^2+^ significantly increases the *E*_a_ value of pathways R2, R3, R4, and R5 (Table 1). Furthermore, the calculation results indicate that the main direct photolysis pathways are not changed by adding Mg^2+^, implying that, for both monomer and complex, pathway R1 (cleavage of N7–C8 bond) is the most easily occurring among the five direct photolysis pathways for NOR^2+^.

For the complex NOR^0^-Mg^2+^, the *E*_a_ values of pathways R1and R2 are higher than those for the monomer NOR^0^, while the *E*_a_ values of pathways R3 and R4 are obviously lower than those for monomer NOR^0^. Mg^2+^ inhibits the cleavage of the N7–C8 bond and C2–C5 bond, but promotes the cleavage of the C10–N13 bond and N13–C14 bond. Furthermore, for the monomer NOR^0^, the cleavage of the N7–C8 bond (pathway R1) is the most easily occurring among the four photolysis pathways, but for the complex NOR^0^-Mg^2+^, the cleavage of the N13–C14 bond (pathway R4) is the most easily occurring.

With the increase of pH, the dominant dissociation species is, in turn, NOR^0^, NOR^+^, and NOR^2+^. As can be seen from Table 1, for de-ethylation (N7–C8 bond cleavage/R1), the order of *E*_a_ is NOR^2+^ < NOR^0^ < NOR^+^, which is not changed by the presence of Mg^2+^. For decarboxylation (C2–C5 bond cleavage/R2), the order of *E*_a_ is NOR^2+^ < NOR^+^ < NOR^0^, which is not changed by the presence of Mg^2+^. For both the loss of the piperazine ring (C10–N13 bond cleavage/R3) and the damage of the piperazine ring (N13–C14 bond cleavage/R4), the order of *E*_a_ is NOR^+^ < NOR^2+^ < NOR^0^, which is changed by the presence of Mg^2+^ (NOR^+^-Mg^2+^ < NOR^0^-Mg^2+^ < NOR^2+^-Mg^2+^). For defluorination (C11–F12 bond cleavage/R5), the order of *E*_a_ is NOR^+^ < NOR^2+^, which is not changed by the presence of Mg^2+^.

## 4. Conclusions

In summary, the computed TS and *E*_a_ indicated that NOR^0^, NOR^+^, and NOR^2+^ showed disparate photochemical reactivity in water. The main photolysis pathways for NOR^+^ were the loss of the piperazine ring via the cleavage of the C10–N13 bond and the damage of the piperazine ring via the cleavage of the N13–C14 bond. The main pathways for NOR^2+^ were de-ethylation via the cleavage of the N7–C8 bond and decarboxylation via the cleavage of the C2–C5 bond. In addition, the presence of Mg^2+^ altered the electronic absorption spectra of NOR and increased the intensities of absorbance peaks of NOR. The TS results demonstrated that Mg^2+^ had dual effects, as it can inhibit or promote the direct photolysis reactions of NOR. Quantum mechanics and density functional theory are potential tools for elucidating the mechanism of photochemical transformation of antibiotics in the water environment.

## Figures and Tables

**Table 1:** Computed activation energies (*E*_a_, kcal·mol^−1^) for the five photolysis reaction pathways (R1, R2, R3, R4, and R5) of three dissociation forms of NOR and complexes with Mg^2+^.

	R1 (Cleavage of N7–C8 Bond)	R2 (Cleavage of C2–C5 Bond)	R3 (Cleavage of C10–N13 Bond)	R4 (Cleavage of N13–C14 Bond)	R5 (Cleavage of C11–F12 Bond)
NOR^0^	21.0	27.6	31.2	26.3	-
NOR^0^-Mg^2+^	23.0	36.5	26.3	18.6	-
NOR^+^	25.4	23.0	9.5	7.7	18.7
NOR^+^-Mg^2+^	27.9	31.2	17.1	12.7	11.4
NOR^2+^	5.4	8.9	30.0	20.5	42.5
NOR^2+^-Mg^2+^	5.2	12.5	35.3	27.3	55.4

“-” represents transition state and corresponding reaction activation energy were not available.

## Supplementary Materials

Supplementary data associated with this article can be found, in the online version.
