# Supplementary material for: Elucidating Direct Photolysis Mechanisms of Different Dissociation Species of Norfloxacin in Water and Mg2+ Effects by Quantum Chemical Calculations
Source: Molecules. 2017 Nov 11;22(11):1949. doi: 10.3390/molecules22111949 (PMC6150356; doi:10.3390/molecules22111949)
Supplement: Supplementary file 1 [file molecules-22-01949-s001.pdf]

# Supplementary Material

# Elucidating Direct Photolysis Mechanisms of Different Dissociation Species of Norfloxacin in Water and $Mg^{2+}$ Effects by Quantum Chemical Calculations

Se Wang* and Zhuang Wang*

Collaborative Innovation Center of Atmospheric Environment and Equipment Technology, Jiangsu Key Laboratory of Atmospheric Environment Monitoring and Pollution Control, School of Environmental Science and Engineering, Nanjing University of Information Science and Technology

* Correspondence: wangse@nuist.edu.cn (S.W.); zhuang.wang@nuist.edu.cn (Z.W.)

5 Pages

4 Figures

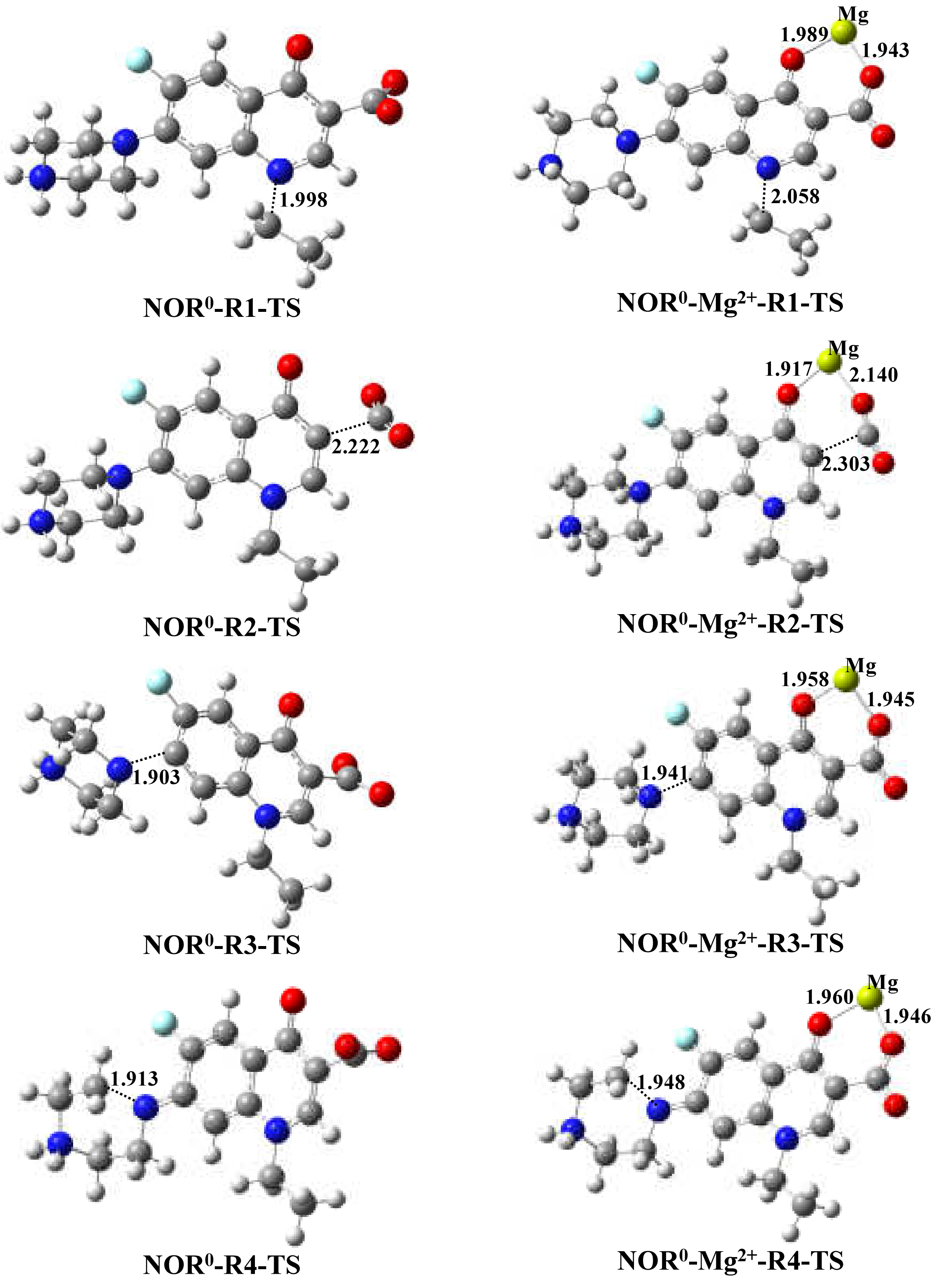


**Figure S1.** Optimized transition state geometries of direct photolysis reaction pathways (R1, R2, R3, and R4) of $NOR^0$ and $NOR^0$-$Mg^{2+}$.

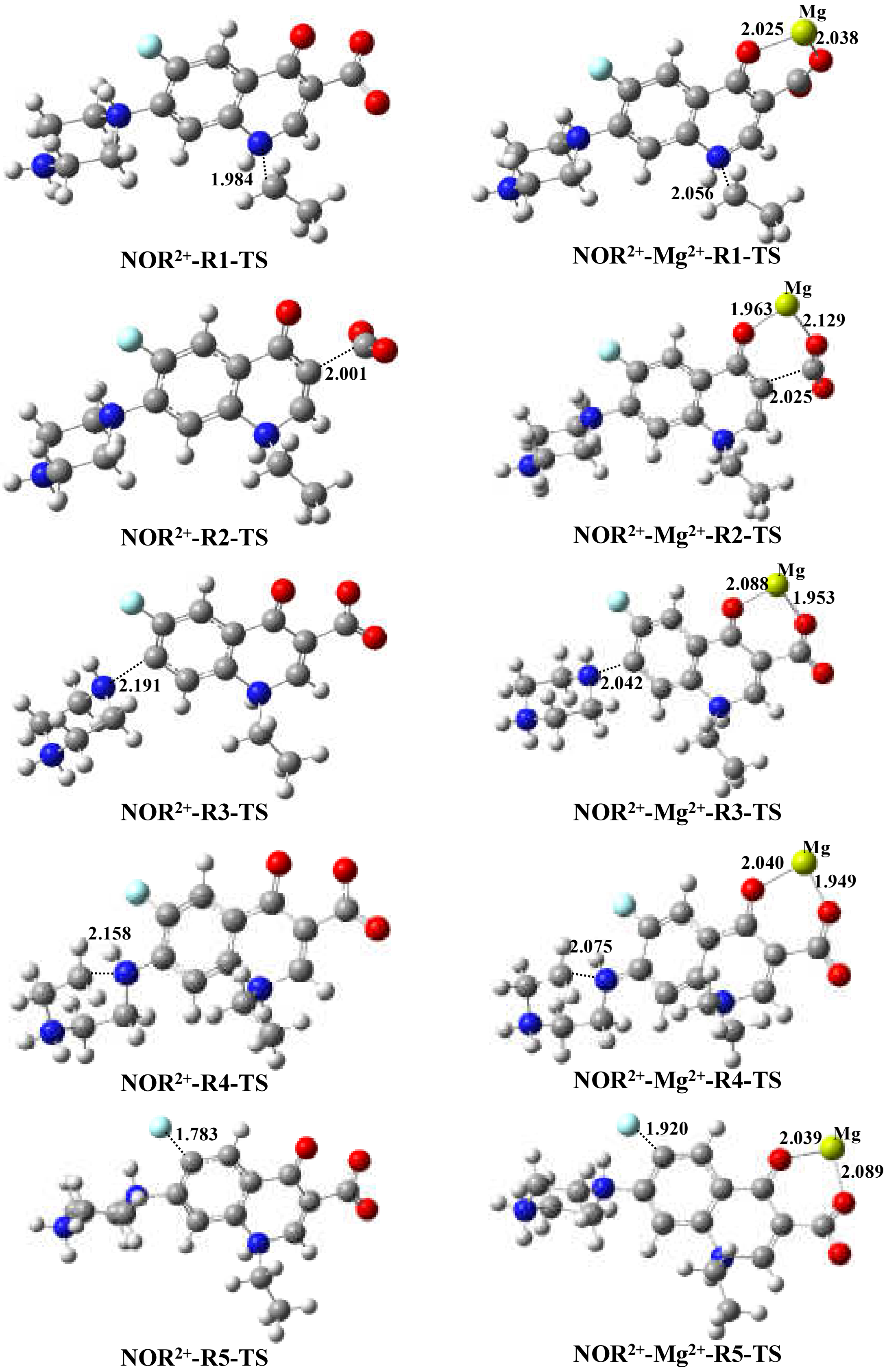


**Figure S2.** Optimized transition state geometries of direct photolysis reaction pathways (R1, R2, R3, R4, and R5) of $NOR^{2+}$ and $NOR^{2+}$-$Mg^{2+}$.

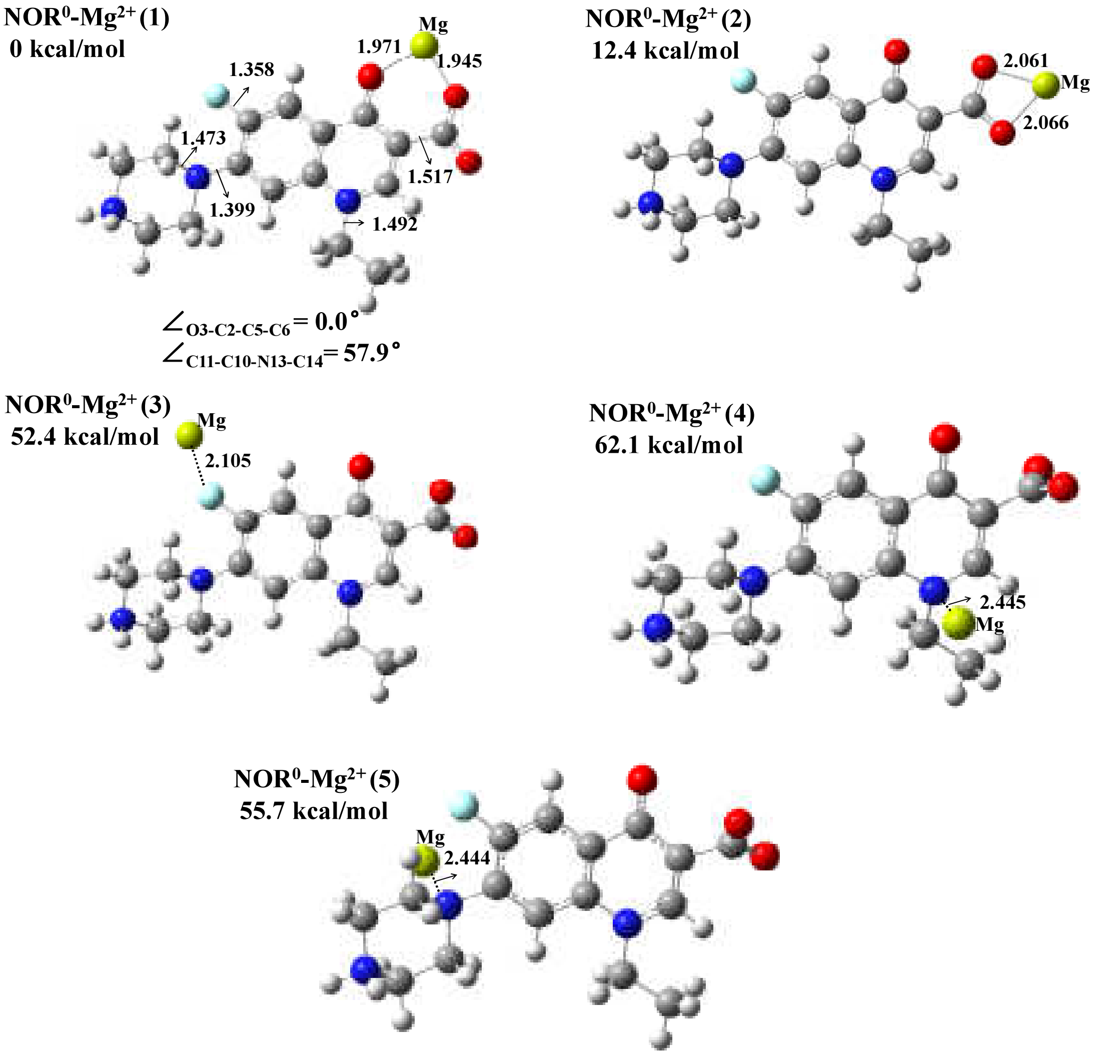


**Figure S3.** Optimized geometries of complex $NOR^0$-$Mg^{2+}$ along with selected bond lengths (Å) and dihedral angles (°). The energies of geometries are relative to that of the most stable geometry $NOR^0$-$Mg^{2+}$ (1).

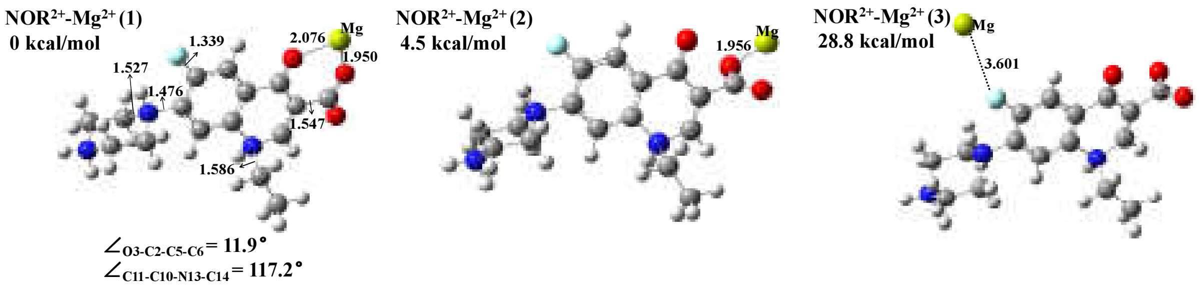


**Figure S4.** Optimized geometries of complex $NOR^{2+}$-$Mg^{2+}$ along with selected bond lengths (Å) and dihedral angles (°). The energies of geometries are relative to that of the most stable geometry $NOR^{2+}$-$Mg^{2+}$ (1).
